# A case report of parapneumonic pleural effusion caused by *Streptococcus pneumoniae* serotype 19A in a child immunized with 13-valent conjugate pneumococcal vaccine

**DOI:** 10.1186/s12887-017-0872-2

**Published:** 2017-04-27

**Authors:** Idrissa Diawara, Khalid Zerouali, Naima Elmdaghri, Abderrahman Abid

**Affiliations:** 1 Laboratoire de Microbiologie, Faculté de Médecine et de Pharmacie, Hassan II University of Casablanca, B.P 5696 Casablanca, Morocco; 2 Service de Microbiologie, CHU Ibn Rochd, B.P 2698 Casablanca, Morocco; 3 Institut Pasteur du Maroc, 1 Louis Pasteur place, Casablanca, Morocco; 4 Service des Maladies Infectieuses Pédiatriques, Hôpital d’Enfants Abderrahim Harouchi, CHU Ibn Rochd, Casablanca, Morocco

**Keywords:** *Streptococcus pneumoniae*, Serotype 19A, Parapneumonic effusion, Pneumococcal conjugate vaccine 13-valent

## Abstract

**Background:**

Simple parapneumonic effusion is a pleural effusion associated with lung infection (i.e., pneumonia). *Streptococcus pneumoniae* remains the most common pathogen causing parapneumonic effusions. In Morocco, the pneumococcal conjugate vaccine 13-valent (PCV13) was introduced in the national immunization program in October 2010 in 2 + 1 schedule for prevention of pneumococcal disease, and replaced by the PCV10 in July 2012 in the same schedule. We report a case of parapneumonic pleural effusions caused by *S. pneumoniae* serotype 19A in a child immunized with 3 doses of PCV13.

**Case presentation:**

This is a 2.5 years old previously healthy Moroccan female, fully vaccinated by PCV13 and immunocompetent, admitted to a private medical clinic with a six months history of persistent asthma. On arrival (7 February 2015), she was febrile to 40.3 °C with a brutal flu syndrome, chills, dry cough and serous rhinitis, for which she received symptomatic treatment. A biological assessment was done that confirmed the clinical diagnosis of flu. Seven days after, she presented a progressive deterioration of its general condition and the onset of severe abdominal pain. She was hospitalized and a second biological assessment, computed tomography scans and chest radiography were done that confirmed a diagnosis of a pneumococcal parapneumonia with abscess of the left lower lobe with encysted empyema. Microbiological analysis of the pleural fluid showed a *S. pneumoniae* serotype 19A with susceptibility intermediate to penicillin. The patient was treated by antibiotics including amoxicillin, cefixime ceftriaxone and vancomycin.

**Conclusions:**

We reported a case of parapneumonic pleural effusions caused by a vaccine serotype pneumococcal 19A occurring in an immunocompetent child immunized with 3 doses of PCV13.

## Background

Simple parapneumonic effusion is defined as pleural effusion associated with lung infection (i.e., pneumonia). Indeed, parapneumonic pleural effusion is a complicated state that occurs subsequently in community-acquired pneumonia. This Complication often necessitates drainage and is associated with prolonged hospitalization [1]. *Streptococcus pneumoniae* remains the most common pathogen causing parapneumonic effusions [2]. In Morocco, the PCV13 was introduced in the national immunization program in October 2010 in 2 + 1 schedule for prevention of pneumococcal disease caused by vaccine serotypes, such as 4, 6B, 9 V, 14, 18C, 19F, 23F (included in PCV7) plus 1, 3, 5, 6A, 7F and 19A. The PCV13 was replaced by the PCV10 in July 2012. Before the PCV introduction, serotypes included in PCV10 and PCV13 covered 71.6 and 82.4% of serotypes associated with IPD among children under five years of age in Casablanca, respectively [3]. However, pneumococcal pneumonia in children or adult is not documented in Morocco. Reports of vaccine failure from PCV13 have been documented most frequently for serotype 3 causing parapneumonic effusion. We reported a case of parapneumonic pleural effusion caused by *S. pneumoniae* serotype 19A in a child immunized with 3 doses of PCV13.

## Case presentation

**Fig. 1:**
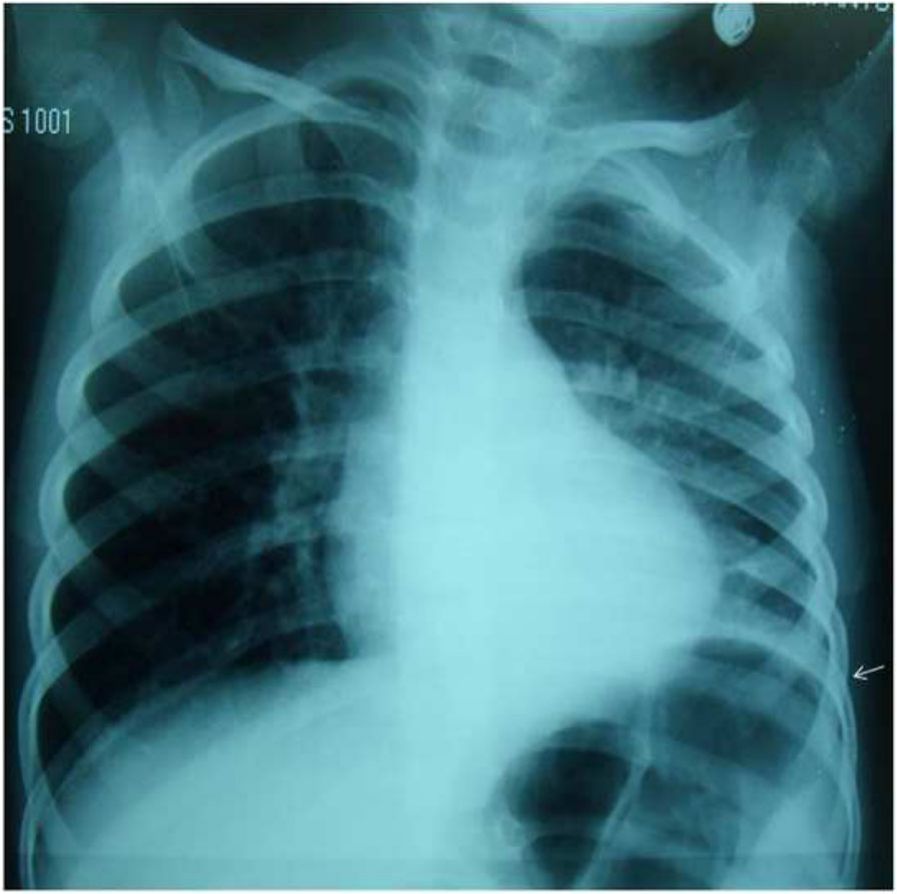
Chest radiograph showed opacity of the left rib base (see *arrow*), erasing the totality of the diaphragmatic cupola filling the pleural cul-de-sac with a pleural band

**Fig. 2:**
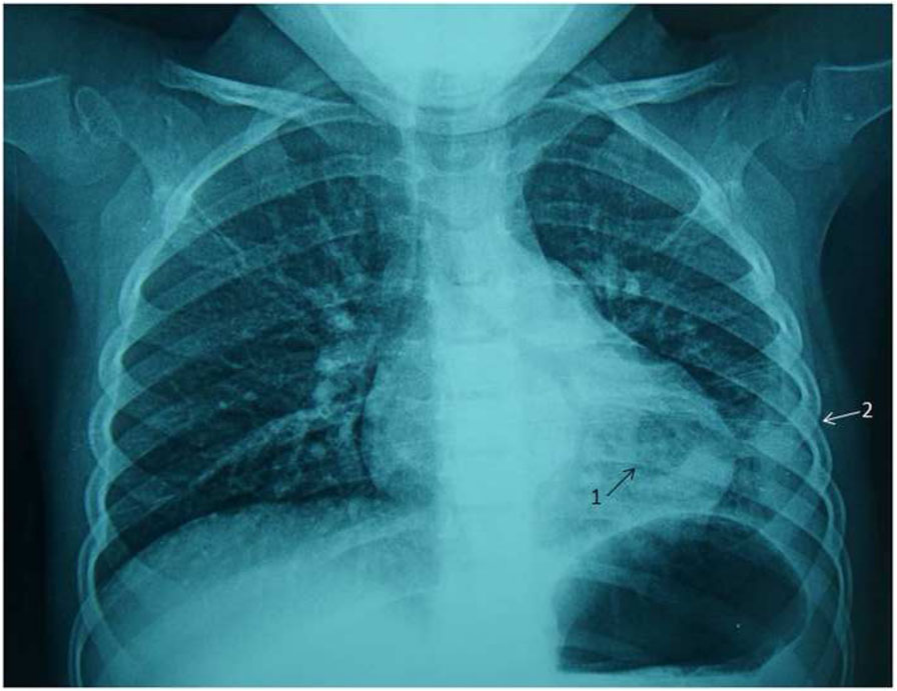
Excavated pneumonia in the lower left lobe (*arrow 1*) with a pleural effusion of 3 cm thick (*arrow 2*)

**Fig. 3:**
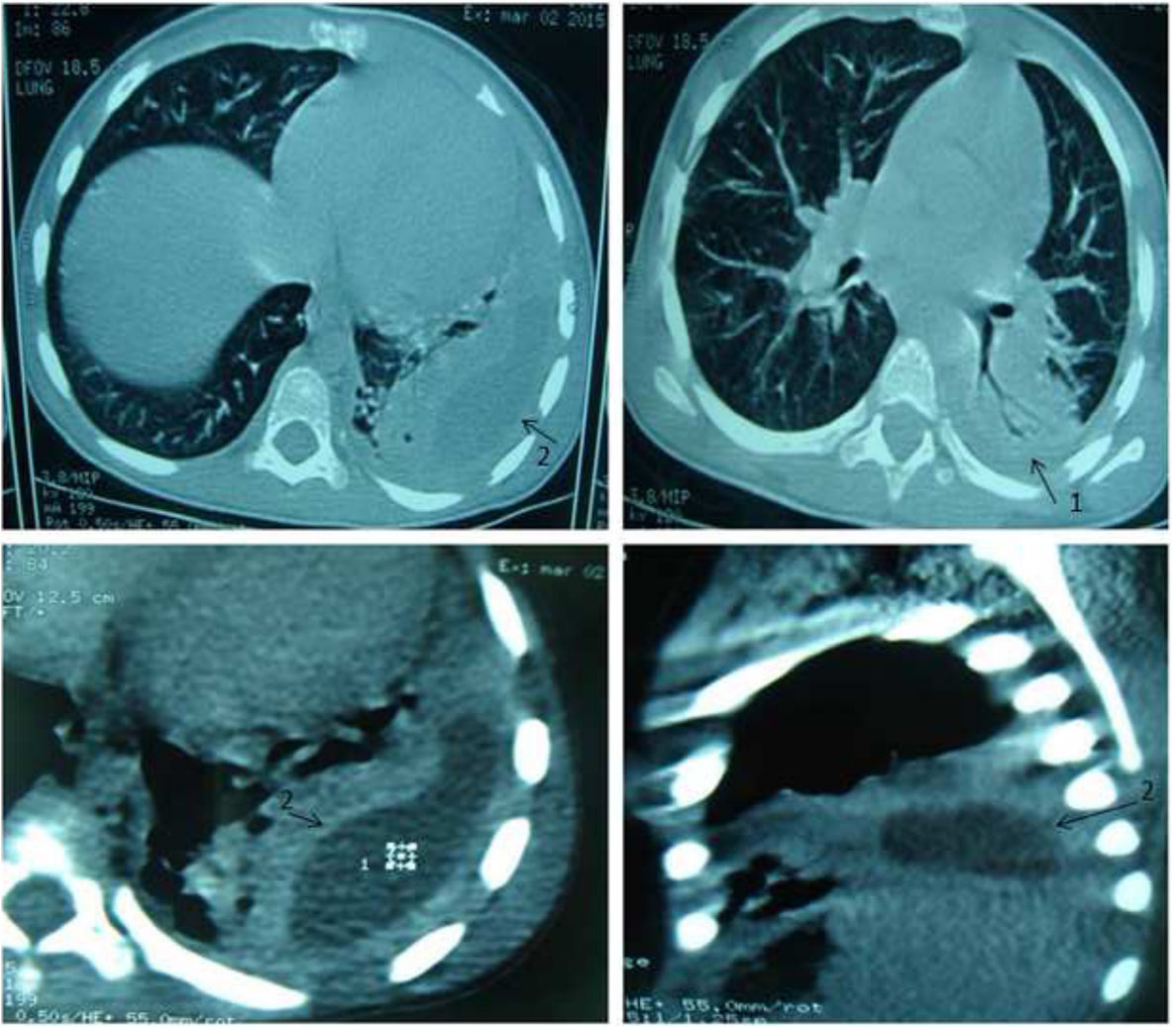
Computed tomography scans of the chest showing a significant parenchymal condensation and shrinking of the left basal pyramid (*narrow 1*) with fluid collection of the convex, suggesting a pleural empyema on the left (*narrow 2*)

A 2.5 years old previously healthy Moroccan female was admitted to a private medical clinic with an antecedent of six months of persistent asthma that was controlled by inhaled corticosteroid therapy. On admission at the private medical clinic (7 February 2015), she was febrile to 40.3 °C with a brutal flu syndromes: chills, dry cough and serous rhinitis, for which she received symptomatic treatment, comprising bed rest and paracetamol. Moreover, she had no dyspnoea, wheezing or crackles on auscultation. After four days, she presented the same symptoms, and a biological assessment was done that confirmed the clinical diagnosis of flu: her white blood cell count was 9540 cells/mm^3^ with 69.7% neutrophils, hemoglobin at 11 g/dL, platelet count at 288,000/mm^3^, C-reactive protein (CRP) was 2.3 mg/L. After seven days, she presented a progressive deterioration of her general conditions and the onset of severe abdominal pain that led to the hospitalization and a second biological assessment was done. According to this biological assessment, her white blood cell count was 17,090 cells/mm^3^ with 85% neutrophils, hemoglobin at 11.1 g/dL, platelet count at 338,000/mm^3^ and the CRP was elevated at 319.28 mg/L. The abdominal-pelvic ultrasound performed in a context of abdominal pain showed no abnormality. A specialized Otorhinolaryngologic examination established the diagnosis of bilateral acute otitis media (AOM), but the child did not complain of earaches and showed no ear discharge. A Treatment with ceftriaxone at 100 mg/kg/day for 3 days was started. On the third day of this treatment, she became afebrile and she left the clinic with the diagnosis of AOM and the treatment of ceftriaxone was replaced by cefixime per os at 8 mg/kg/day every 12 h for four days. At home, because she developed once again a rapid fever, cefixime was replaced by amoxicillin/clavulanate at 80 mg/kg/day every 8 h for seven days, but this changing did not provide any favourable clinical result. However, chest radiography of front that was made in these hospital records was initially misinterpreted and was wrongly considered as normal. Thereafter, an extensive analysis of this first chest radiograph showed an opacity of the left thoracic base, erasing the totality of diaphragmatic cupola and filling the pleural cul-de-sac with a pleural band of 1 cm thick (Fig. 1). Moreover, the child has begun having evident difficulties to breath with a chest pain, dyspnoea and expiratory moaning. A second chest radiography was performed after thirteenth day of disease. The result of this radiography rectified the diagnosis and showed an excavated pneumonia in the lower left lobe with a pleural effusion of 3 cm thick (Fig. 2). Finally, a chest computed tomography scan confirmed the diagnosis of abscess pneumonia with empyema (Fig. 3).

After 23rd day of the disease, the absence of apyrexia despite amoxicillin/clavulanate treatment and the need to identify the etiologic agent, the child was re-hospitalized and a pleural puncture under general anaesthesia was performed at two intercostal spaces (5th axillary and 6th posterior). The 5th pleural space allowed to withdraw more than 6 mL of pus and sent to the laboratory for analysis.

Laboratory analysis was performed at the Microbiology Laboratory of Ibn Rochd University Hospital Centre of Casablanca. This analysis showed a diplococci positive Gram in direct examination and bacterial culture revealed the presence of *S. pneumoniae*. Serogrouping was done by the checkerboard method with Pneumotest-latex (Statens Serum Institute antisera, Copenhague, Denmark) and serotyping was performed by Quellung capsule swelling using Statens Serum Institute antisera (Copenhague, Denmark). These methods of serogrouping/serotyping classified the serotype as 19A. Antibiotic susceptibility testing was done following the Clinical Laboratory Standard Institute guidelines (CLSI, 2015). Erythromycin, tetracycline, choramphenicol, vancomycin, levofloxacin, rifampicin and cotrimoxazole were tested by disk diffusion; penicillin and ceftriaxone by E-test on Mueller Hinton Agar supplemented with 5% sheep blood. The strain was susceptible to vancomycin, chloramphenicol, rifampicin and levofloxacin; intermediate susceptibility to tetracycline, ceftriaxone (MIC = 2 μg/mL) and penicillin (MIC = 4 μg/mL); and resistant to erythromycin, and cotrimoxazole. Quality control of antibiotic susceptibility testing was conducted using *S. pneumoniae* ATCC 49619.

**Fig. 4:**
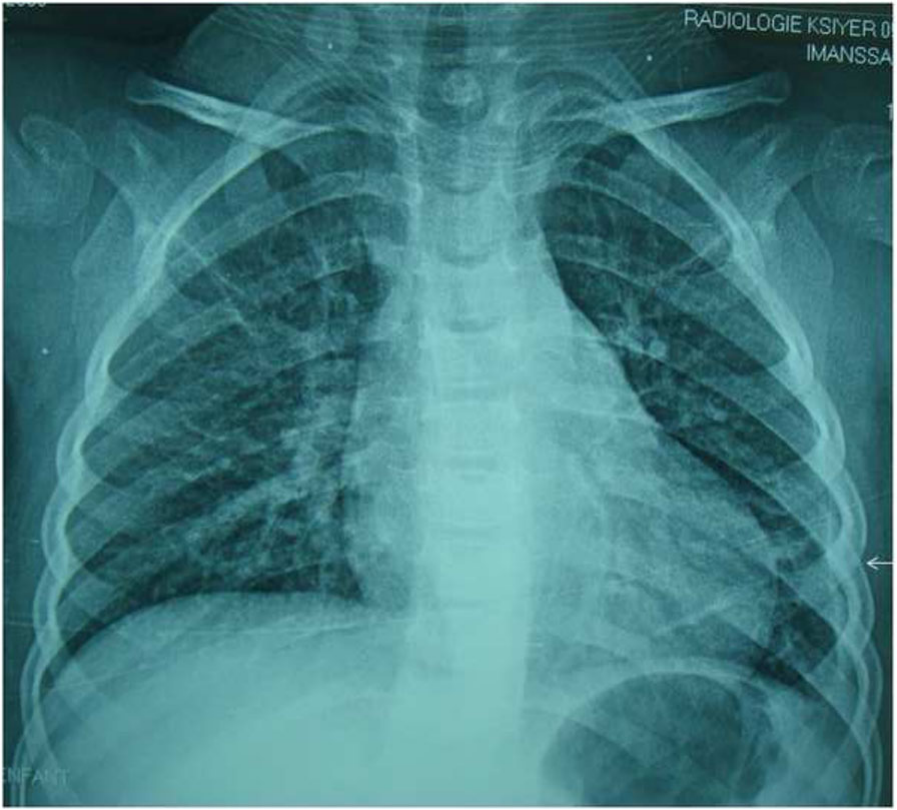
Chest radiograph showing the cleaning of pneumonia and empyema (see *arrow*)

The final diagnosis of the disease was a pneumococcal pneumonia with abscess of the left lower lobe with encysted empyema: a pneumococcal parapneumonic pleural effusion. The treatment with amoxicillin/clavulanate was stopped and replaced by ceftriaxone at 100 mg/kg/day by slow intravenous injection and vancomycin 60 mg/kg/day by continuous infusion. After 10 days of these treatments, she continued as outpatients with ceftriaxone in the same doses and teicoplanin by slow intravenous injection in a single dose by day. After eight days of these treatments, she was apyretic, her white blood cell count was 4420 cells/mm^3^ with 15% neutrophils, hemoglobin at 10.7 g/dL, platelet count at 308,000/mm^3^, and CRP was normal at 0.6 mg/L. The pneumonia was completely treated as showed on chest radiography (Fig. 4). After two weeks (24 march 2015), antibiotic treatments were stopped.

The examination of her vaccine status showed that she received PCV13 vaccine at 2 months of age, 4 months of age and the third dose at 12 months of age (2 + 1 doses). The development of IPD despite the complete vaccination, led us to undertake an immune deficiency evaluation three months after the disease (on June). This assessment revealed that the total classic complement pathway screen was within normal range and Immunoglobulin (Ig) Gamma and its subclasse, IgA and IgM levels were normal. Furthermore, she has not presented any high risk for invasive *S. pneumoniae* disease in children included chronic heart disease (particularly cyanotic congenital heart disease and cardiac failure); diabetes mellitus; cerebrospinal fluid leak; cochlear implant; sickle cell disease and other hemoglobinopathies; anatomic or functional asplenia; HIV infection; chronic renal failure and nephrotic syndrome.

## Discussion

In a previous study, we reported data about IPD from children less than five years of age in Casablanca [4]. We showed that after vaccine implementation, the main IPD that affected children were meningitis, bacteremia and some rare cases of pleural infections. The examination of vaccine status showed that 40% of vaccine serotypes were isolated from children who received at least one dose of PCV10 or PCV13 in Casablanca. Vaccination schedule was incomplete for all vaccine failures described in this last study [4]. Furthermore, the replacement of PCV13 by PCV10 vaccine in the Moroccan NIP could affect serotype distribution at national scale, because PCV10 does not contain serotypes 3, 6A and 19A. Elsewhere, Vaccine coverage of PCVs vaccines in children aged to 2 years of age was estimated to 88% at the Grand Casablanca in 2014 as declared by the observatory regional of epidemiology service of health of Casablanca. In the present case report, we reported a pneumococcal parapneumonia in a child that previously presented a flu infection associated with an AOM. Influenza infections can make some people more susceptible to develop bacterial pneumonia. *S. pneumoniae* is the most common cause of this bacterial pneumonia among children. Pneumococcal infections can be a serious complication of seasonal influenza infections [5].

Pneumococcal infections are mainly caused by the colonizing serotypes and are therefore more susceptible to develop antibiotic resistance [6]. Moreover, antibiotic pressure has a continuing role in increasing pneumococcal resistant strains in respiratory infections. Pneumococcal serotype 19A remains a redoubtable serotype because of its clinical implication of pneumococcal disease and its ability to develop higher level of antibiotics resistance [7].

As for the diagnosis of flu, it was based mainly on clinical diagnosis because the child presented the majors clinical symptoms of flu and the first biological assessment showed no sign of inflammation in favour of bacterial infection.

Concerning the management of empyema, in most cases, it is managed by drainage [8]. For the case presented here, due to the low abundance of pus in the empyema, we did not practice the pleural drainage. We adopted the strategy of the use of antibiotic therapy alone without drainage, nor early decortications. The persistence of fever for more than 10 days remains for us a classical data in a context of empyema of the child.

Parapneumonic effusion and empyema are disease processes that evolve over time and involve different management strategies [8]. To prevent pneumococcal diseases, WHO recommended the inclusion of PCVs in childhood immunization programmes worldwide [9]. Surveillance after the introduction of a PCV to the standard childhood immunization schedule demonstrated that the vaccines were effective in preventing IPD and providing herd immunity [10]. In the United States after the introduction of PCV to the standard childhood immunization schedule, the overall rate of pneumococcal pneumonia has declined but the absolute number of cases of complicated pneumococcal pneumonia and the proportion of invasive pneumococcal disease caused by pneumonia had increased [11].

Pneumococcal antibody titers vary over time, even in healthy subjects. With the change of vaccine strategy, waning coverage can occur for serotypes that not include in the new vaccine. Vaccine failure has been reported in fully immunized children, due to serotype 3 in the parapneumonic pleural effusion context after 3 + 1 doses of PCV13 [12], but also serotype 19A in bacteremic pneumococcal pneumonia after 3 + 1 doses PCV13 [13]. There are currently no reports of PCV13 failure in preventing complicated pneumonia caused by serotype 19A among children vaccinated with the full 2 + 1 schedule. These reported cases highlighted that pneumococcal serotypes can differ in their abilities to colonize the nasopharynx, and in whether they are likely to cause invasive disease once they do colonization. The serotypes can differ also in what disease manifestations (e.g., conjunctivitis, otitis media, pneumonia, bacteremia, or meningitis) they are more likely to cause and with what complication, whether they are likely to be resistant to antimicrobial agents or to escape vaccination, where they are most predominant geographically [7].

It is typical when describing vaccine failures, to verify whether or not the PCV13 was administered correctly with the correct formulation, no storage issues with the doses, on the correct dates according to the child’s age. According to the information provided by the paediatrician who vaccinated the child, the three doses of PCV13 have been correctly administered in the antero-external face of the thigh. Moreover, the vaccine complied with the recommended cold chain.

A potential limitation of this case report is that we were not able to determine the pneumococcal serotype 19A IgG level for the patient during her illness and after disease.

## Conclusions

This is the first documented case of parapneumonic pleural effusions caused by vaccine serotype 19A occurring in an immunocompetent child immunized with 3 doses of PCV13. Further surveillance is necessary to see how the effectiveness of PCV10 vaccine will reduce IPD caused by non-PCV10 vaccine serotypes.

## Abbreviations

AOM: Acute otitis media
CRP: C-reactive protein
IPD: Invasive pneumococcal disease
MIC: Minimum inhibitory concentration
PCV10: Pneumococcal conjugate vaccine 10-valent
PCV13: Pneumococcal conjugate vaccine 13-valent
PCV7: Pneumococcal conjugate vaccine 7-valent
